# Actual conditions of person-to-object contact and a proposal for prevention measures during the COVID-19 pandemic

**DOI:** 10.1038/s41598-022-22733-9

**Published:** 2022-10-27

**Authors:** Teruaki Hayashi, Daisuke Hase, Hikaru Suenaga, Yukio Ohsawa

**Affiliations:** Department of Systems Innovation, School of Engineering, The University of Tokyo, 7-3-1, Hongo, Bunkyo-ku, Tokyo, 113-8656 Japan

**Keywords:** Environmental social sciences, Health care

## Abstract

This study focused on human contact behavior with objects and discussed countermeasures during the COVID-19 pandemic across 15 location types. Reducing contact with objects and disinfecting items can be implemented at a relatively low cost. We created a protocol for organizing the objects, and 1260 subjects who went outside during a day between December 3–7, 2020 in Tokyo and Kanagawa, Japan were surveyed. The participants touched 7317 objects in total; the most common objects were doors, chairs, baskets, elevator equipment, and cash. One-way analysis of variance and Scheffé’s multiple comparison test showed that supermarkets had the lowest mean and median values despite having the highest number of users, contact objects, and object types. Conversely, the values for hotels were the highest, significantly higher than that for other places, excluding amusement parks, workplaces, and schools and universities. Furthermore, the long-tailed frequency distribution of the number of objects suggests that the objects touched by many individuals are limited; thus, it is important to determine the objects to be prioritized for disinfection at each location. The data and protocol could inform infection countermeasures that properly address the contact realities as they pertain to people’s behavior and objects.

## Introduction

The novel coronavirus disease (COVID-19) caused by severe acute respiratory syndrome coronavirus 2 (SARS-CoV-2) is currently spreading worldwide, resulting in widespread infection. Although SARS-CoV-2 is primarily transmitted through the air^1,2^, the complete picture of its transmission remains unclear^3^. In addition to direct cases, indirect infection via public restrooms or elevators or both has been suspected^4^, while the risk associated with having an infected person in the same space has been reported^5^. Several laboratory experiments have found SARS-CoV-2 RNA on the surfaces of objects such as computer mice, trash cans and their handles, patients’ bedframes, doorknobs, and restroom amenities^6–8^. The possibility of infection through contact with a surface onto which an infected person coughed or sneezed has not been ruled out, especially if contact occurs before the virus dies. SARS-CoV-2 has been demonstrated to be highly stable on the skin^9^ and to survive on paper currency, cell phone screens, and stainless steel in the dark^10^. The World Health Organization (WHO) states that the virus can survive for up to 72 h on plastic surfaces and up to 24 h on cardboard^11^. Although some have argued that infection from the surface of objects is rare^12,13^, frequent disinfection of surfaces and objects that have been touched by multiple individuals is important.

Rather than waiting for a vaccine to become widely available, non-pharmacologic interventions for infection spread prevention have been implemented^14–21^. In particular, hand sanitization and reduced contact with objects are among the most accessible infection control measures that can be implemented at a relatively low cost. Therefore, it becomes important to understand the objects that are frequently touched by people (hereafter referred to as “contact objects”) and which objects need to be prioritized for disinfection. However, it may be extremely difficult to disinfect all such items. In previous studies, doorknobs, elevator buttons, or trash can handles have been selected and observed arbitrarily and empirically from among a myriad of other objects; however, the specific contact objects investigated should differ depending on the location and personal behavior. From this perspective, a comprehensive understanding of contact objects is lacking.

Accordingly, the purpose of this study was three-fold. First, we aim to investigate the actual status of human-object contact behavior. Second, to examine the objects with which people come into regular contact based on activity type. Third, to outline measures to be taken at each location by discussing the risk of infection through inanimate surfaces. As with studies on the simulation of infection control^22^ or measurement of household water insecurity^23^, this study did not investigate infected individuals or SARS-CoV-2 RNA directly but provided useful insights into COVID-19 control and prevention measures.

In this study, 1260 individuals living in Tokyo and Kanagawa prefecture, Japan, participated in a survey, which was used to collect and analyze participants’ behavior patterns and the objects they touched on days that they went out of their respective households. The findings of this study are expected to provide data that could improve our understanding of actual human behavior and contact with objects, which in turn could lead to more effective infection countermeasures. Such data are also expected to contribute to the proposal of disinfection prioritization during periods of rapid infection spread.

## Methods

### Subject demographics

This study was conducted using a two-stage online survey comprising a preliminary survey and a main survey. The two-stage survey was conducted by targeting residents of Tokyo and Kanagawa prefecture, which are the most populous areas in Japan. These regions were also experiencing an increased infection spread as of December 2020 (as of December 2020, 60,177 and 21,262 infections were reported in Tokyo and Kanagawa, respectively). The spread in these specific areas accounted for 34.5% of the total number of infection cases in Japan. Tokyo, the capital of Japan with a population of approximately 14 million people, is also the country’s center of politics, economy, and culture. Kanagawa prefecture, in turn, is adjacent to the southern areas of Tokyo and is the second most populous city after Tokyo (approximately 9 million people). The prefecture’s capital is the city of Yokohama.

### Study design

The participants were asked to respond, in detail, to a survey regarding the locations they stayed at for an extended period between December 3 (Thursday) and December 7 (Monday), 2020, and all the items that they touched during this time. The participant behavior was found to be as diverse as the types of contact objects. Therefore, using the locations where clusters of infections were found during April 2020, as reported in Refs.^4,24,25^, 12 locations were selected (e.g., medical facilities, including hospitals; restaurants; stores whose main objective was to sell alcohol, such as bars; companies, including the participants’ own workplaces and that of others; and sports facilities such as gyms) and investigated. Similarly, three means of transport, namely trains, buses, and taxis, were selected as spaces where people often crowd. The detailed items obtained from the survey data, such as the locations where the participants spent the most time, as well as the number of people with whom they spent time during the research period, are described in Supplementary Information Sections 1.1 and 1.2.

The participants were asked to provide information regarding the locations where they spent most of their time during the corresponding period. They were also asked to detail all the objects they touched (excluding personal objects) during this time. The objects in this study were evaluated using a free-writing description. Typographical errors and differences in expressions were frequently observed (e.g., water closet, toilet, and bathroom). A categorization rule was thus developed to better ascertain the actual status of locations and object contact. The participants’ expressions were modified through visual inspection, and the objects were organized to be separated into three entities: space, object, and component. “Component” refers to attachments or parts (e.g., buttons, levers, and knobs).

### Patient and public involvement

This survey was conducted after appropriate review by the Ethics Committee of the Graduate School of Engineering, the University of Tokyo (examination number: 20-61; approval number: KE20-72). Informed consent for study participation was obtained from each participant, and the collected data were coded and the analysis was performed with an anonymized database. All methods were performed in accordance with the relevant guidelines and regulations.

First, 143,464 residents of Tokyo and Kanagawa prefecture, consisting of both men and women (20–69 years old), were initially asked to cooperate in the preliminary survey on November 27, 2020. Participants for the main survey were then narrowed down to 1943 participants who consented to the study and indicated a specific intention to cooperate. To improve the accuracy of the survey, it was confirmed during the preliminary survey that the participants had plans to leave their households during December 3 (Thursday) to December 7 (Monday), 2020 (i.e., the research target days). The participants were then asked to provide the dates and locations to which they were planning to go. The main survey was conducted with 1536 subjects during December 3–7, 2020. Data from 1260 subjects who gave valid responses were used for the analysis. To ensure that the participants could respond while their memories were still fresh, the survey was distributed to each participant on the day of their corresponding behavior.

### Protocol for organizing the object entities

**Figure 1:**
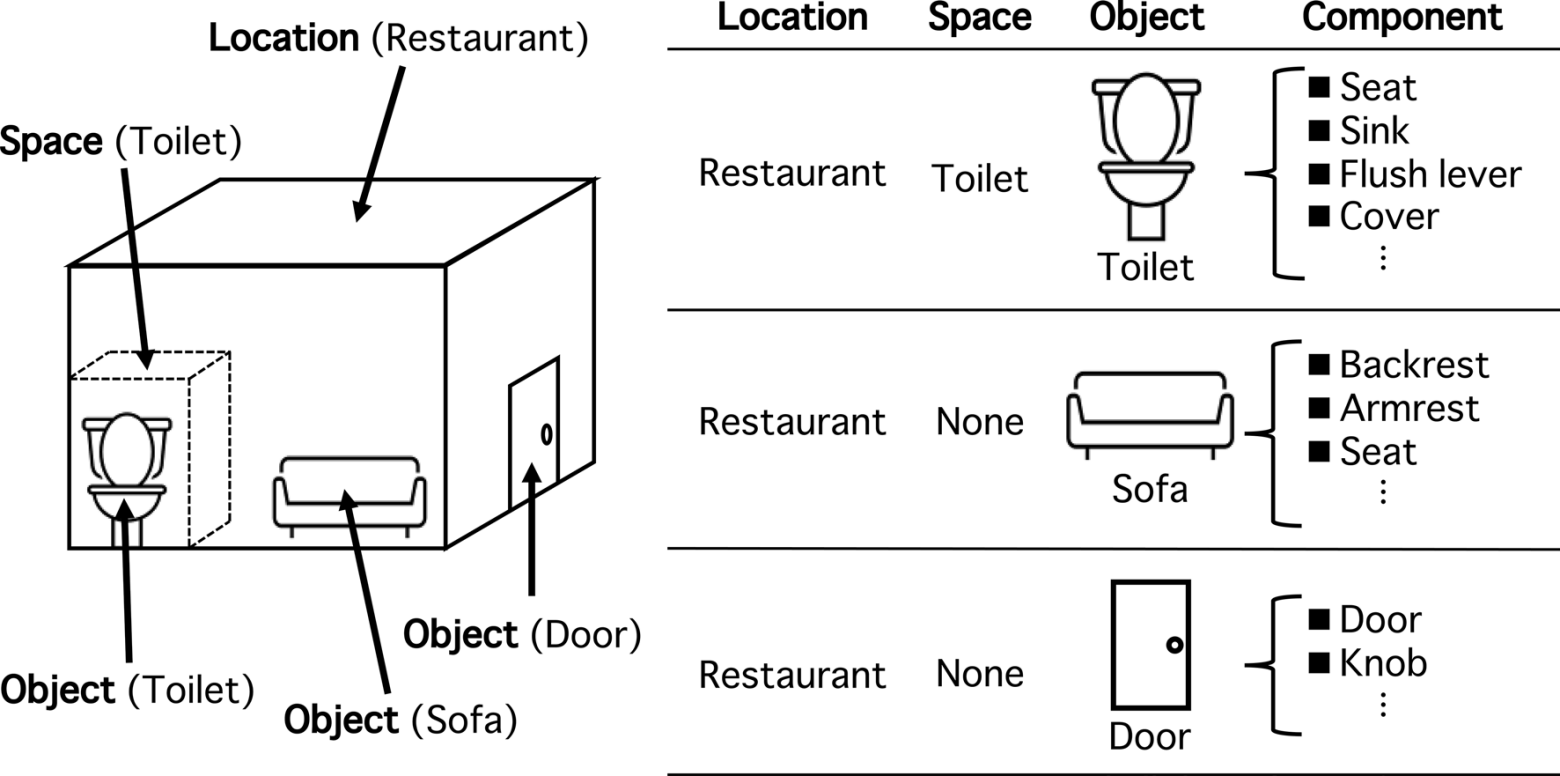
Scheme for organizing objects in this study. “Location” was a major category that contains several “spaces.” The figure on the left shows an example of a restaurant, which is one of the 12 major locations investigated in this study. The restaurant contains three objects, sofa, door, and toilet. In the example, toilet is a space (space: toilet) that contains the object toilet (location: restaurant; space: toilet; object: toilet). The figure on the right is an example of the structure of the objects included in the space and their components. As “space” was often omitted, such entries sometimes did not exist (expressed as “none”).

The participants were asked to respond about the locations where they spent most of their time during the corresponding period and to detail all the objects they touched during this time, excluding personal objects. The object data in this study were collected using a free-writing description. We developed a categorization rule to better ascertain the actual status of locations and object contact. Participant expressions were modified through visual inspection, and the objects were organized in order to be separated into three entities: space, object, and component (Fig. 1).

**Figure 2:**
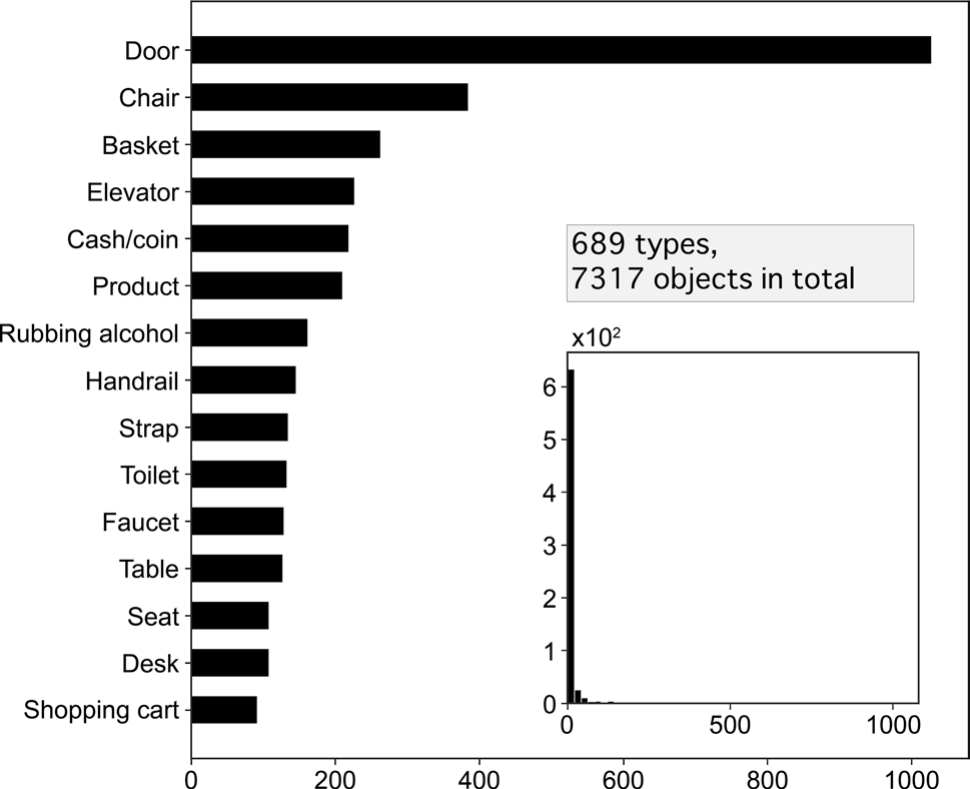
Distribution of the 15 most touched objects (bins: 50). There were 689 types of objects, and a total of 7317 object items were touched. The histogram (bins: 50) has a structure in which objects with a small occurrence frequency account for the majority of the total occurrence frequencies (i.e., 323 types of objects with an occurrence frequency of 1, and 94 types of objects with an occurrence frequency of 2).

**Figure 3:**
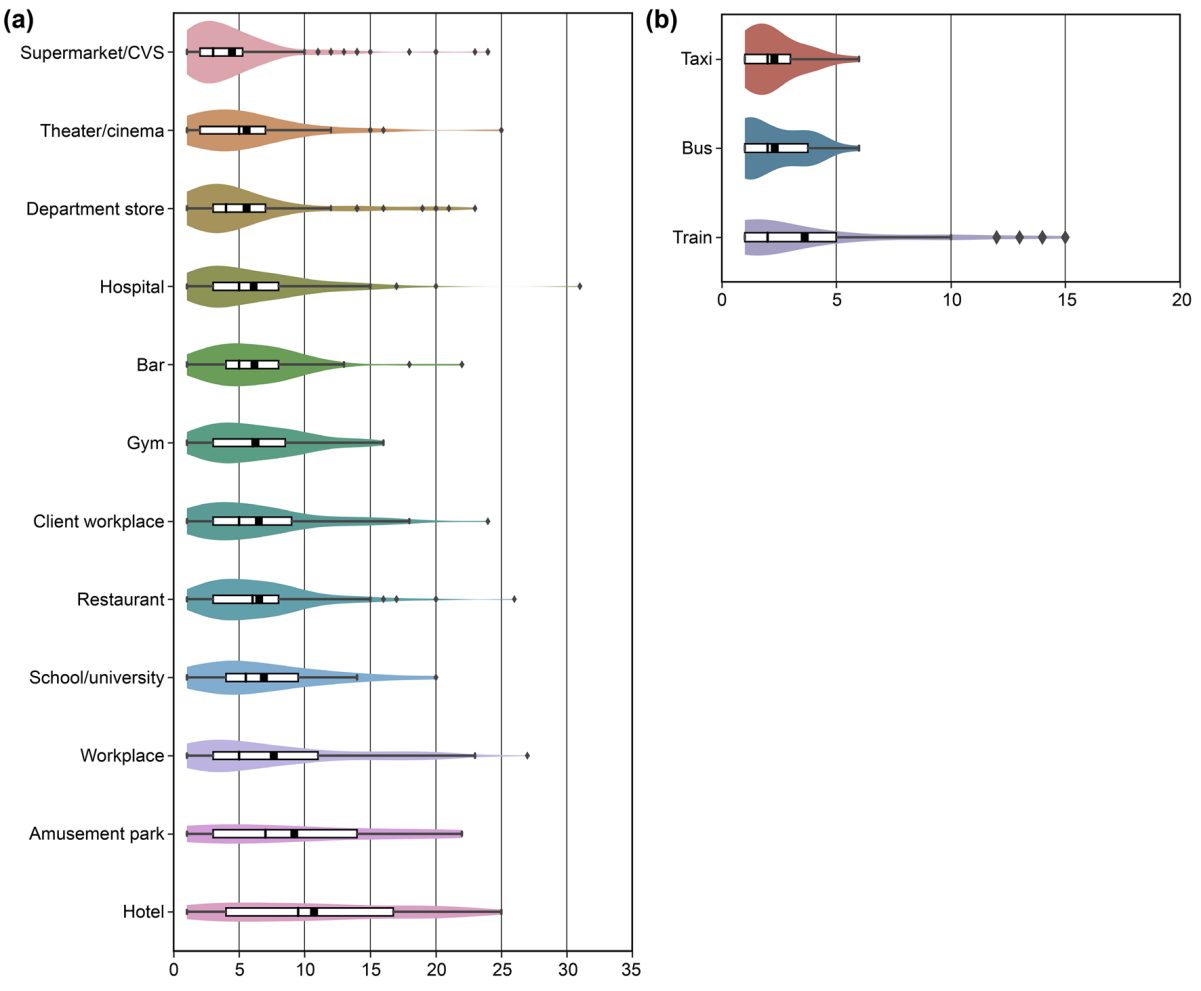
Box-and-whisker plots showing the number of objects that were touched (**a**) at each location and (**b**) per vehicle type. Here, the objects are placed in descending order according to their mean values. The left end of the white box represents the first quartile and the right end represents the third quartile, while the left end of the whisker represents the first quartile $-1.5\times IQR$ and the right end of the whisker represents the third quartile $+1.5\times IQR$. The black square within the white box represents the mean value, and the black bar represents the median value. Diamonds indicate outliers. Each box-and-whisker plot is accompanied by a distribution curve.

**Figure 4:**
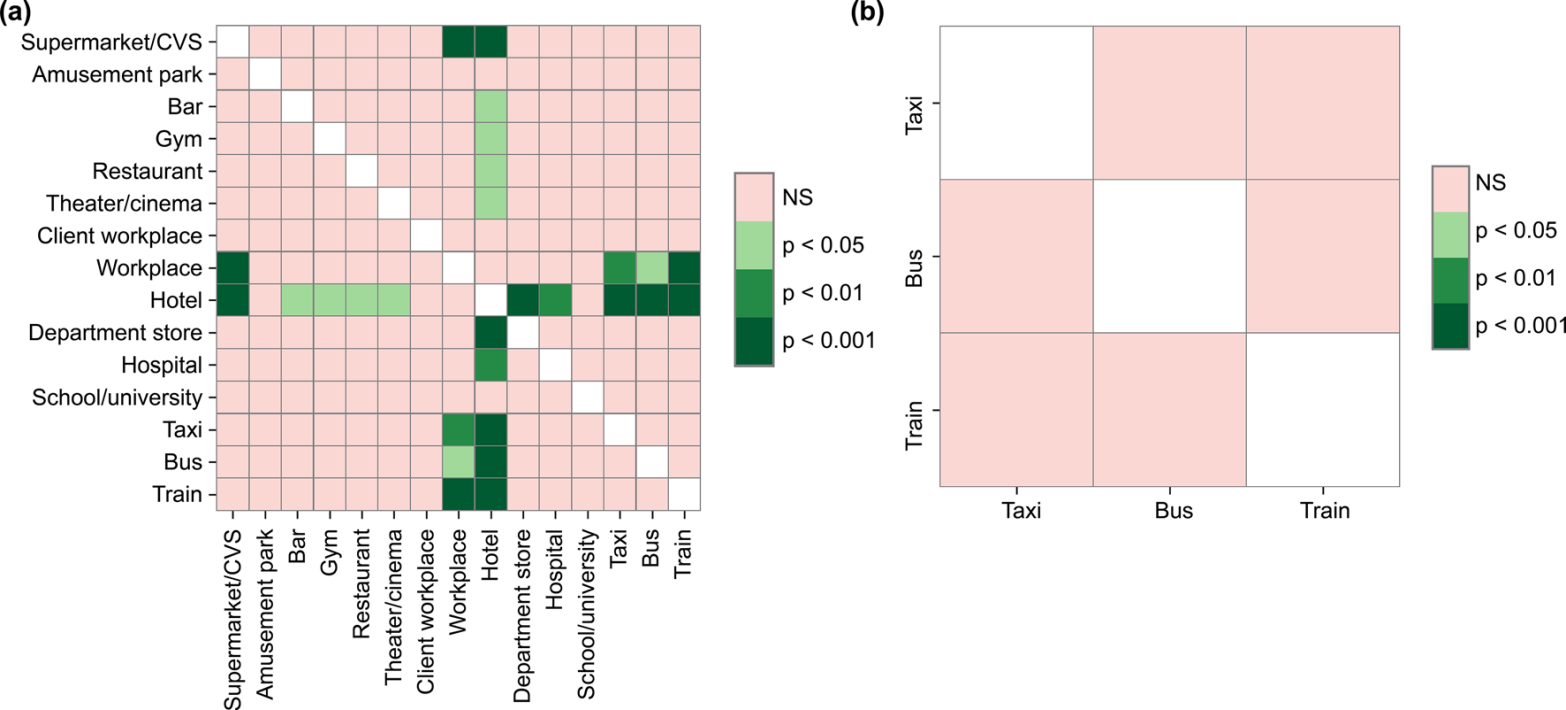
Heat maps of multiple comparisons of the number of contact objects at (**a**) each location and (**b**) each vehicle using the Scheffé test. To determine significant differences between groups in terms of the number of objects with which subjects came into contact at each location, a multiple comparison using the Scheffé test was conducted.

**Figure 5:**
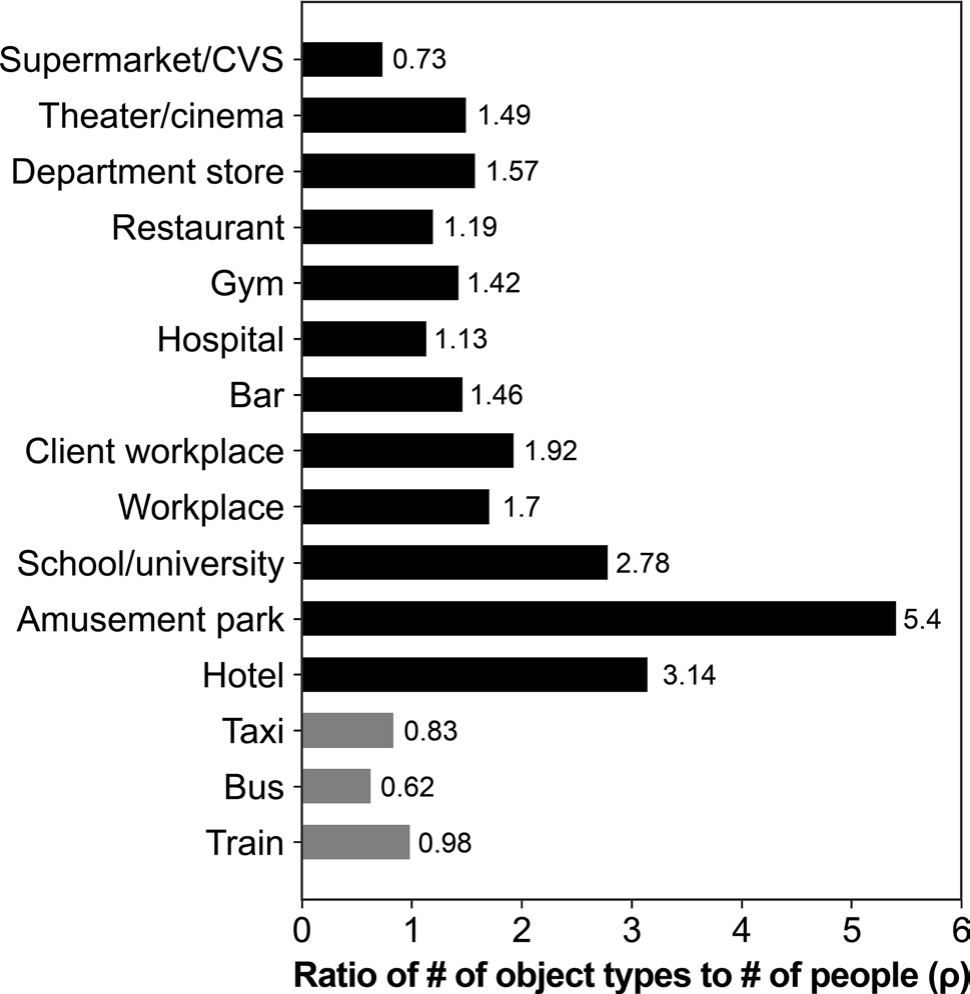
Values for each location/vehicle when the ratio of the object type to the number of users is expressed using $\rho$. Higher ratios indicate an increase in the type of objects that can be touched per person in a given location, which means that more objects would need to be disinfected. In contrast, low ratios indicate few types of objects per person. Thus, many individuals may touch the same objects, and the number of objects that need to be disinfected is limited.

**Figure 6:**
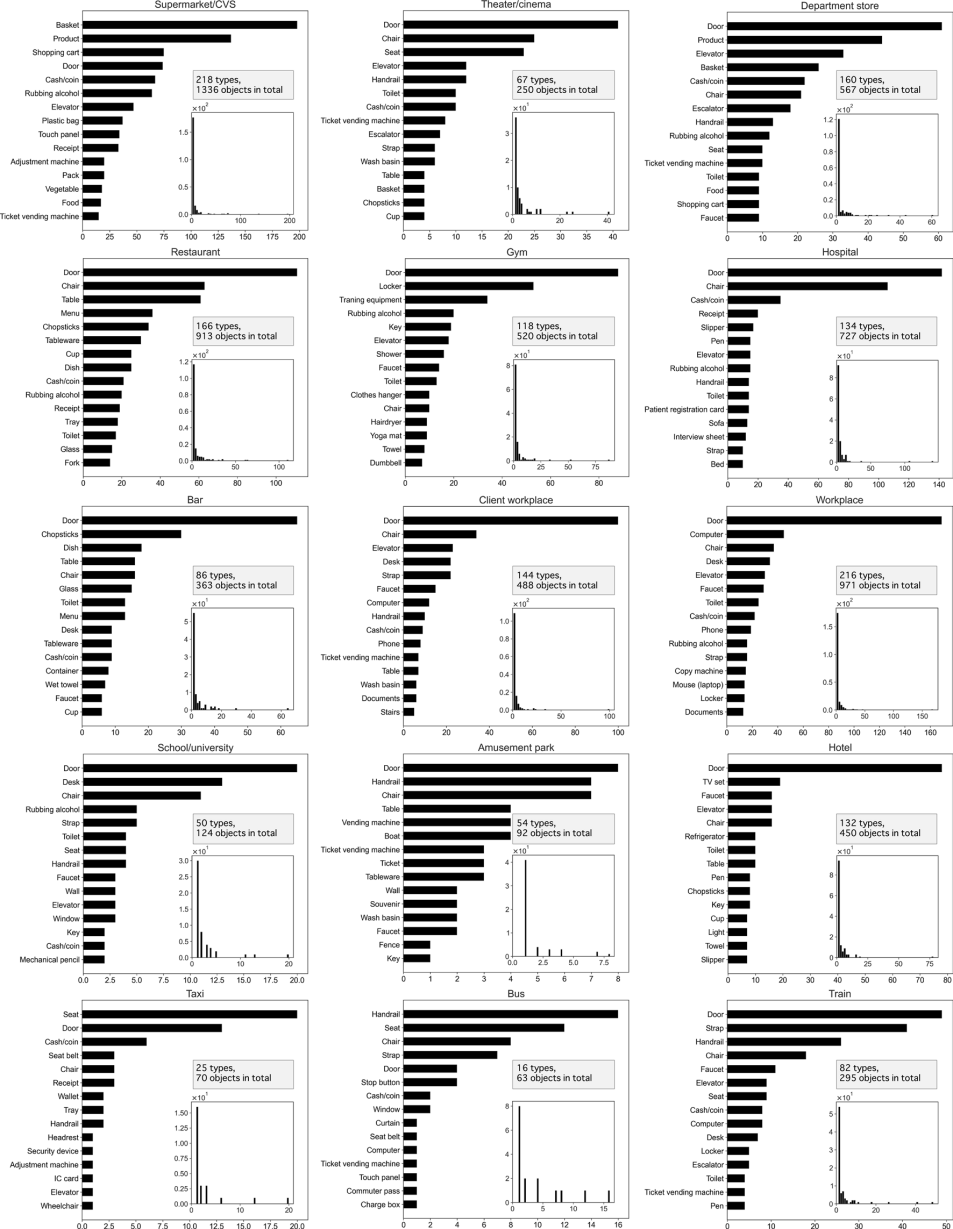
Top 15 contact objects at the 12 locations/three vehicles and occurrence frequency histograms (bins: 50). A structure in which objects with a small occurrence frequency accounted for the majority of the total occurrence frequencies appeared for all locations and vehicles. Doors were the most touched object in all locations except in supermarkets/CVSs.

Objects always have the entity of an “object;” thus, free-writing descriptions of “space” and “component” were often omitted. For example, in the case of a refrigerator door, the refrigerator would be the “object” and the door would be the “component.” Conversely, with a restroom door, the restroom would be the “space” and the door would be the “object.” Such difference is the result of the refrigerator door forming a part that is attached to the rest of an object (i.e., the refrigerator), while the restroom door is a door associated with a “space” (i.e., the restroom).

As a result, a major “location” item exists in addition to “space,” “object,” and “component.” “Location” comprises the 12 locations and three vehicles, as presented in Table S4. As seen with {location: train; space: train; object: strap}, “location” often overlaps with “space,” and “space,” at times, is omitted. The protocol for organizing the entities was as follows:

1. Typographical errors were confirmed and corrected by three people (including the authors of this paper) by reading the responses.
2. Items divided with a comma and the word “and” were treated as separate objects, e.g., “cash and credit card” → {object: cash}, {object: credit card}.
3. Synonyms and objects with the same concepts were unified, e.g., “toilet,” “bathroom,” and “WC” → {object: toilet}.
4. Product and store names were unified with a general name, e.g., “Takashimaya” → {space: department store} and “Asahi beer” → {object: beer}.
5. Objects for which the location was clearly described were separated between locations and objects, e.g., “changing room door” → {space: changing room; object: door}.
6. If there were several locations, multiple “spaces” would be set, e.g., “toilet faucet at a golf course shop” → {space: golf course; space: shop; space: toilet; object: faucet} and “refrigerator door in the office waiting room” → {space: office; space: waiting room; object: refrigerator; component: door}.
7. Long descriptions were abbreviated, e.g., “bag distributed at the theater” → {space: theater; object: bag} and “purchased clothes” → {object: clothes}.
8. Objects for which the component(s) were clearly described were separated between components, e.g., “flush lever of the toilet” → {object: toilet; component: flush lever} and “armrest of the sofa in the restaurant” → {space: restaurant; object: sofa; component: armrest}.
9. Japanese responses were translated to English.

The object with the highest number of components was the seat. Seats comprise six components, namely, seat belts, backrests, recliners, seats, seat covers, and handrails. This object was followed by the toilet, which is composed of the following five components: toilet seat, bidet, button, lever, and lid. Seats also have components such as chairs and backplanes; however, these were not included in the data as they were not directly touched.

### Statistical analysis

One-way analysis of variance (ANOVA) and Scheffé’s multiple comparison test were performed for each of the 12 location groups to compare whether there were differences in mean values for each location and vehicle data group. The significance thresholds for this analysis were 0.001, 0.01, and 0.05. The results of a Tukey–Kramer test, conducted as a reference, are described in Supplementary Information Section 2.1. As a vehicle is a space different from a place (location), the analysis results for this factor are discussed separately. A dedicated environment for statistical analyses was created using Python’s scikit-posthocs (v. 0.6.7).

## Results

### Actual contact behavior in relation to objects

The survey results established that the 1260 participants touched a total of 7317 objects across 15 types of locations and vehicles, with objects spanning 689 different types (806 types if their separate components were included). Figure 2 shows the top 15 most touched objects. The most common items touched by the participants were doors, chairs, baskets, elevator equipment (buttons, doors, and handrails), and cash (i.e., in and from cash registers). The inset in Fig. 2 shows a histogram of the occurrence frequency distribution of the top objects (bins: 50). This histogram exhibits a long-tail distribution in which objects with a low occurrence frequency accounted for the majority of types, with objects with occurrence frequencies of one to five times accounting for 523 types (76.0% of the total). The number of high-contact-frequency objects was smaller. The same tendency was observed in the analysis of object components (Supplementary Information Section 2.2).

Given that SARS-CoV-2 is often detected on trash can and door handles in commercial establishments^7^, infections via building and room doors may occur. This is because there are many opportunities for multiple, unspecified individuals to touch these doors, including infected individuals. In particular, given that hospital restroom doorknob samples tested positive on PCR tests^6,8^, it can be assumed that doors are one object type that should be disinfected regularly and intensively. Conversely, chairs and shopping baskets have been rarely mentioned in previous studies, even though they are objects of frequent contact. In addition, considering that the survival rate of viruses is considered to be high on objects such as cash^10^, it is necessary to pay attention to the transfer of cash and related actions. For example, the present study found that cash was touched 219 times: receipts, 90 times; IC cards (e.g., credit cards), 51 times; and ticket vending machines and credit card payment machines, 142 times. Objects that hold the potential to be touched regularly assume an exponential distribution close to a long-tail distribution. It should be noted that doorknobs and cash used in previous studies were arbitrarily and empirically chosen from a myriad of other objects. Doorknobs are important objects for disinfection because they are generally touched frequently. However, testing for SARS-CoV-2 RNA may be necessary not only for arbitrarily selected locations but also for objects that are touched regularly, as partially identified in this study.

From the viewpoint of disinfecting objects, the types and number of objects that an individual touches can be very large, suggesting that disinfecting all such items could be expensive. However, the distributions demonstrated in this study revealed that items with a low occurrence frequency account for the majority of the total occurrence frequency. For example, if an object was reported to have been touched by more than 100 people, it was considered to be an item that is touched by a large, unspecified number of individuals. The total number of such objects was 14, a manageable number for disinfection purposes.

### Locations/vehicles and touched objects

Next, we compared contact objects according to the location and vehicle where the participants spent the longest time on a corresponding day. The overall trend was that the number and types of contact objects increased linearly as the number of users increased (Supplementary Information Section 2.3). Notably, supermarket/convenience store (CVS) had the lowest mean and median values among the 12 locations, despite having the highest number of users, contact objects, and object types (Fig. 3a). This means that while the number of supermarket/CVS users is high and the types of objects are varied and numerous, the contact frequency with any particular object per person is very low. However, hotels had a relatively small number of users (42 people), 132 object types, and a total of 450 contact objects. While such numbers are not large, the mean and median values for hotels were 10.7 and 9.50, respectively, both of which were the largest among the 12 locations. Thus, the number of objects touched per person in hotels is high compared to that in other locations.

An analysis of variance was used to determine the differences in mean values for each data group at each location. The ANOVA results indicated significant differences across the data of the 12 location groups (f-value $=9.03$, $p<0.001$). Next, multiple comparisons were performed using the Scheffé test, a rigorous test to determine significant differences between groups (Fig. 4a). The mean value for hotels was significantly higher than that for other locations, excluding amusement parks, workplaces, and schools and universities, all of which had comparatively high mean values. It follows that the number of contact objects per person for the average hotel user was higher than the average for the 12 locations. According to Supplementary Information Section 2.4, the length of the hotel stay was not correlated with the number of contact items. Therefore, it can be concluded that the mean number of contact objects in hotels is high, regardless of an individual’s length of stay.

Conversely, in the case of vehicles, the number of commuters using trains was the highest (84), while buses and taxis had less than half the number of commuters. Similar to the locations noted previously, a linear increase was seen in both the number and types of objects touched when the number of users was high. Although the mean for trains was higher than that for buses and taxis, the median values were virtually the same (Fig. 3b). An analysis of variance was performed for the three vehicle groups, similar to the one-way ANOVA conducted for the aforementioned locations. However, in this case, the groups or number of contact objects did not differ significantly (f-value $=2.51$, $p=0.084$), and no significant differences were observed using the Scheffé test (Fig. 4b). Nine out of 84 train commuters touched an extremely large number of objects, becoming outliers. Train-related spaces are composed not only of stations but also multiple spaces, such as cafes and restrooms. Therefore, individuals who used these spaces increased the mean contact objects for train commuters.

The ratio of the types of objects to the number of users was set to $\rho$ (Fig. 5). The large number of types of objects for the number of users indicate the great variety of objects that can be touched at any given location. In other words, higher ratios indicate an increase in the types of objects that can be touched per person in a given location, which means that more objects would need to be disinfected. However, this finding also means that there are fewer opportunities for one object to be touched by multiple individuals. Thus, a higher ratio indicates more types of objects that are available to be touched; however, fewer people will be touching the same object. Conversely, a low ratio means that there are many opportunities for multiple individuals to touch the same object because the number of object types per person is small. In this case, fewer objects need to be disinfected.

The highest $\rho$ was established at amusement parks (5.40), followed by hotels (3.14) and schools and universities (2.78). Supermarkets/CVSs (0.72) had the lowest ratio, followed by hospitals (1.13) and restaurants (1.19). Regarding amusement parks, hotels, and schools and universities where the ratio was high, the number of objects available to be touched in the corresponding location was also high. Therefore, many objects will need to be disinfected. However, comparatively fewer objects can be touched by multiple individuals at these locations. Conversely, supermarkets/CVSs, hospitals, and restaurants have many objects that are touched regularly, which means that objects in such locations should take disinfection priority. In places where there are simply a large number of users, the density results in an increase in the risk of infection. In this regard, avoiding places with many users is an important measure. However, locations with high $\rho$ values-places with few users but many contact objects-are where users share the same spaces (locations and vehicles), both directly and indirectly, which increases the chances of contact with an unspecified number of people. From the perspective of remaining within an inter-community to prevent infection spread^22^, we should avoid these places and refrain from contacting the objects there.

Figure 6 presents histograms of the top 15 contact objects, as well as the contact frequency of objects in each location and vehicle (bins: 50). For the 12 locations and three vehicles, the histograms present a structure in which the occurrence frequency of objects with low contact frequency is high. In other words, a few objects are touched by all users at a high frequency, and most of the objects are touched by only a limited number of individuals. Such findings may seem negative, considering that the objects that need to be disinfected vary; however, such findings are also positive, as discussed in the previous subsection. The long-tail structure of the frequency distribution means that the objects touched by many and unspecified individuals are limited; thus, it is important to determine the objects to be prioritized for disinfection in each location.

## Discussion

### Measures for contact with objects at each location

In this section, the measures for each location and vehicle are discussed. Additionally, Supplementary Information Section 2.5 discusses restrooms and doors, which had a particularly high occurrence frequency and have often been noted in previous studies.

### Supermarkets/CVSs and department stores

Shopping is an essential activity for ordinary citizens; accordingly, the number of users for supermarket/CVS was the highest at 300. Therefore, both the total number of objects (1336) and object types (218 types) were inevitably the largest among the different categories. The mean (4.45) and median (3.00) values were, however, the smallest among the locations, and the contact frequency with objects was very low (Fig. 3a). These findings indicate that supermarket and CVS users know what they want to buy; therefore, they do not engage in unnecessary object contact. At department stores, the number and types of objects that can be touched are lower than at supermarkets and CVSs, and both the mean (5.56) and median values (4.00) were slightly higher than those for supermarkets and CVSs. However, the $\rho$ of department stores was 1.57, which is twice as high as that of supermarket/CVS (Fig. 5). As discussed in the previous section, a higher ratio indicates that the types of objects that a person can come into contact with at the corresponding location will be higher. Therefore, for effective disinfection at department stores, a larger number of objects will need to be disinfected. Conversely, for supermarket/CVS, the $\rho$ was the lowest at 0.73, indicating that more objects were regularly touched by people. In Fig. 6, shopping baskets, general merchandise, shopping carts, and doors ranked high in both supermarket/CVS and department stores. As a large, unspecified number of individuals come into contact with these items, frequent disinfection is required.

In addition, supermarket/CVS had the highest number of outliers at 24, followed by department stores with 12 outliers. These results indicate that retailers are characterized by a large number of customers who touch an extremely large number of items. Therefore, it should be noted that while many users touch only a few objects, attention should be paid to the large number of users who touch many objects. Furthermore, both supermarket/CVS and department stores display many products that have a high contact frequency, e.g., merchandise, food, and fresh produce. These include products that are picked up by hand but not purchased, as well as items displayed as samples. Thus, in addition to guidelines for food safety and COVID-19^26^, guidelines may also be required for products that are touched but not purchased. Cases of cluster infections have been reported in shopping malls in which no direct contact occurred. Therefore, it is crucial to disinfect doorknobs and elevator buttons as these are touched by many unspecified individuals^4^.

### Hotels and amusement parks

The number of hotel users, including accommodation facilities such as Ryokans (a traditional Japanese inn) and hostels, was small at 42, possibly because the study was conducted during a period of widespread infection when travel was restricted. Nevertheless, the mean number of contact objects per person was the largest at 10.7, with the median being high at 9.50. Amusement parks, which are sites associated with travel similar to hotels, also had a low number of users at 10. However, the mean number of contact objects was 9.20, which was the second-highest after that of hotels. This finding could be attributed to increased contact opportunities with objects that individuals do not normally touch at places that provide experiences outside of the ordinary (e.g., travel and leisure). Furthermore, the $\rho$ in hotels and amusement parks was very high, 3.14 and 5.10, respectively. Most of the items in hotels that were touched frequently were in guest rooms, such as TV sets, keys, lights, and air conditioners (Fig. 6). However, while the variety of items was high, these items are not generally touched by a large, unspecified number of individuals. Furthermore, although many objects need to be disinfected, the proportion of objects touched by multiple individuals was also found to be the lowest.

In Japan, a tourism policy, known as the “Go To Travel Campaign,” was enacted in July 2020 to help the tourism industry, which was negatively impacted by the COVID-19 pandemic. This campaign involves the movement of many people, which, in turn, would signify the movement of infected individuals. For this reason alone, the opportunities for many people to come into contact with even more objects increased. Travel-related COVID-19 cases possibly increased during the early days of the campaign^27^. As $\rho$ was very high, the objects touched per person were varied. In addition, opportunities to have contact with objects as well as sharing a space with a variety of people with whom one would not usually come into contact (i.e., at the travel destination) will increase. Therefore, extra attention is necessary to effectively disinfect these locations and their related objects.

### Restaurants and bars

Among restaurants and bars, restaurants had slightly higher mean and median values. However, this difference was not statistically significant (Fig. 4a). The distribution of objects with which users came into contact had a similar structure. Notably, restaurants have more types of tableware and seasonings, and the number of object types that people generally touched was double, with the total number of objects touched being nearly three times as high. Overall, because the top contact objects in Fig. 6 were similar and $\rho$ was slightly higher for bars, the measures to be taken are generally the same.

Both aerosol and contact infections are of particular concern in food and drink settings. Although the effectiveness of face coverings (i.e., masks) has been noted in various studies^20,21^, actions such as taking off face coverings are common in places where eating and drinking take place. The increased risk of infection in settings associated with eating and drinking has been noted in various previous studies^1,28^. The virus reportedly remains active on stainless steel, heat-resistant glass, and plastic for several hours^9^. If SARS-CoV-2 survives on the skin and other surfaces, the risk of infection through contact with such surfaces is high.

In the survey, places where food- and drink-related objects were served included restaurants, bars, and theaters/cinemas (e.g., drinks and food for watching movies), and food- and drink-related activities (e.g., having lunch in dining halls) occurred in client workplaces, workplaces, schools and universities, hotels, and hospitals, whereas eat-in meals were reported for department stores. In these locations, the removal of masks is expected; therefore, effective ventilation is required, and disinfection of hands and objects with frequent contact occurrences should be thorough.

### Workplaces and client workplaces

No significant difference was found between the participants’ workplaces and client workplaces (Fig. 4a). However, slightly more objects were touched in the participants’ workplaces (Fig. 6). Although the number of object types in participant’s workplaces was 216, or 1.5 times more than in client workplaces, the $\rho$ for each space was similar at 1.70 and 1.92, respectively. Thus, there were no location reports with extremely high numbers of objects. Visiting client workplaces inevitably leads to more opportunities for people to come into contact with a large, unspecified number of other individuals and objects with which they might not normally come into contact. In either place, doors, computers, chairs, desks, and elevators have the most opportunities to be touched, and therefore, these objects should be prioritized for disinfection.

### Schools and universities

Although the number of users for schools (including universities and vocational schools) was low, at only 18 students, the mean value was the third highest at 6.88. In addition, $\rho$ was 2.78, which was also the third highest, indicating that schools are places with a high diversity of objects considering the number of users. However, the occurrence frequency of objects other than doors, tables, and chairs was low, which leads to the conclusion that the number of objects that should be prioritized for disinfection is low.

### Hospitals

Hospitals had the fourth-highest number of users (119), with a moderate number of contact objects (134 types). Moreover, hospitals had the second-lowest $\rho$ (1.13), after supermarket/CVS. Hospitals also contained many objects that were regularly touched by individuals. Doors and chairs were particularly common contact objects because hospitals consist of multiple spaces, each of which is occupied by an examination chair or a chair in a waiting room.

It should be noted that hospitals function as medical sites while simultaneously having the potential risk of cluster infections. Therefore, a variety of studies have been conducted on this topic. For example, an air sample taken 4 m from a patient tested positive using PCR. Although the presence of infectious viruses was inconclusive, samples from computer mice, trash cans, patient bedframes, doorknobs, and even the cuffs and gloves of medical practitioners were found to be PCR-positive^6^. In hospital wards in Singapore, the virus was detected on bathroom doorknobs and toilet bowl surfaces using PCR tests^8^. In another reported case from Wenzhou, China, indirect infection via restrooms and elevators was suspected without direct contact being confirmed^4^. Unventilated hospital restrooms can also become hotspots for infection^19,29^. Specifically, SARS-CoV-2 has been detected in fecal-originating aerosols in hospital restrooms^30^. As highlighted by Ding et al., periodic disinfection of toilet surfaces and doorknobs is essential for limiting the spread of infection.

### Theaters/cinemas

Doors, chairs, and seats occupied the upper positions in theaters/cinemas. In addition, theaters/cinemas had the least movement among the 12 locations studied. Therefore, theaters/cinemas had the second-lowest mean among the 12 locations, with only 67 types of contact objects. Although the number of contact objects was small, theaters/cinemas involve actions accompanied by eating and drinking, which leads to similar issues as in restaurants and bars. Therefore, precautions similar to those for restaurants and bars should be applied in theaters/cinemas.

### Gyms

Gyms are characterized by a large number of spaces, including not only exercise areas but also pools, showers, restrooms, and changing rooms. Doors and lockers accounted for a high percentage of total contact objects in these places. The $\rho$ was relatively low because many of the objects in gyms are shared (e.g., training equipment and lockers). As seen in the case of an infection outbreak at a gym^25^, attention should be paid not only to object contact but also to social distancing, as there are multiple spaces with the potential for crowding, such as locker and shower rooms and training areas. Similar to eating and drinking activities, it is expected that people will remove their masks for long periods in gyms, indicating that measures are needed to ensure good ventilation.

### Trains, busses, and taxis

Regarding the vehicles studied (i.e., trains, buses, and taxis), trains were found to have the largest number of users, types of contact objects, and mean values. As opposed to taxis and buses, trains have many contact objects, including ticket gates, ticket vending machines, seats, hand straps, handrails, and other people’s body parts (in cases of congestion and crowding). In addition, as mentioned previously, trains have many spaces (e.g., restrooms attached to stations). This type of transport is also characterized by many individuals who touch an extremely high number of objects. However, the $\rho$ values for all vehicles were less than unity. This finding suggests that vehicles tend to contain a large number of objects that are regularly touched by many people. Therefore, objects such as seats, handrails, and doors should be prioritized for disinfection.

### Limitations and future issues

Several issues need to be addressed in future work. First, as the focus was on general locations, such as hospitals, supermarkets/CVSs, hotels, and others, the data did not include facility information, such as location-specific objects or population density. Accordingly, we will focus on specific facilities, conduct fixed-point observations, and trace contact with objects in future work.

Second, as the data are related to the current coronavirus pandemic and were collected in December 2020, changes in contact behavior as compared to those before the pandemic could not be tracked. Importantly, the spread of COVID-19 varies according to season, weather, and event^31–33^, and the precautionary behaviors depend on individual characteristics such as gender, age, and employment status^34^.

Third, this study was conducted in Japan’s urban areas, which experienced (and continues to experience) the rapid spread of the virus. However, the spread of infection differs depending on the density of the cities in question and general congestion^35^. In addition to the findings of this study, it is necessary to discuss the exact characteristics of the objects and target locations to determine their suitability to future outbreaks of infectious diseases. For example, while Western-style toilets are standard in Japan, squatting toilets are found in many parts of the world. Furthermore, Japan has a relatively high level of trust in the sanitation of public spaces, and small public spaces characterize the high population density of Tokyo and Kanagawa prefecture. Cultural and social backgrounds might have a significant impact on the relationship between objects and contact behavior.

Fourth, regarding the locations, it is expected that the frequency and type of contact objects will vary by occupation type. We believe that tracking not only the location but also the purpose of the visit will lead to a more detailed understanding of the dynamics of object contact.

Finally, there are limitations associated with the survey data. As this study aimed to examine contact with objects at each location, we could not obtain the detailed attributes of objects and sequence of actions before and after the contact. The order of objects touched during eating and drinking and the action of bringing the hand to the mouth may provide important clues to identifying more microscopic routes of infection. Moreover, doors are fixed to a room, but cash, keys, and cups, for example, are movable objects. The movability of objects would provide further insight into the likelihood of unspecified individuals touching them and determine their disinfection priority.

## Conclusion

In this study, actual conditions of people’s contact behavior in relation to objects were ascertained. The relationship between these behavior conditions and various contact objects was analyzed, and we discussed the types of contact that vary according to locations and vehicles, the corresponding infection risks, and possible countermeasures. Although this study did not address the objects with which COVID-19 patients came into contact, the data could still inform infection control policies that properly address the realities of contact as they pertain to people’s behavior and objects. Therefore, the data are believed to contribute to the general principle of disinfection prioritization during periods of widespread infection. In particular, operators and individuals in the locations discussed in this paper should engage in prioritized disinfection and avoidance of contact behavior with regard to objects.

## Supplementary Information

Supplementary Information.

## Data Availability

The datasets generated and analyzed during the current study are available in the Kaggle dataset repository, https://www.kaggle.com/teruakihayashi/person-to-object-contact-dataset.
