# Supplementary material for: Actual conditions of person-to-object contact and a proposal for prevention measures during the COVID-19 pandemic
Source: Sci Rep. 2022 Oct 27;12:18092. doi: 10.1038/s41598-022-22733-9 (PMC9610312; doi:10.1038/s41598-022-22733-9)
Supplement: Supplementary file 1 — Supplementary Information. [file 41598_2022_22733_MOESM1_ESM.pdf]

Actual Conditions of Person-to-Object Contact and a Proposal for Prevention Measures During the COVID-19 Pandemic

SUPPLEMENTARY INFORMATION

1. Data outline

1.1 Research data items

Table S1 presents the data acquired from the preliminary and main surveys. Items in bold letters are those used in this study for the analysis. The industry classification presented in this study was based on the Japanese Standard Industry Classification (revised November 2007), as stipulated by the Ministry of Internal Affairs and Communications (Table S2). Since there is no industry classification for subjects who are homemakers, students, or unemployed, their occupations were specified as homemakers, students, or unemployed, respectively.

Table S1. Data items obtained in the preliminary survey and main survey (items in bold letters were used in this paper)

Preliminary survey	Main survey
Sex, age group, area of residence (municipality), respondent's occupation, industry classification, residential type (e.g., detached housing or apartment housing), unmarried, composition of cohabiting family, household type (using the Ministry of Internal Affairs and Communications standards), household annual income, the planned outing date, and behaviors of the day, accompanying person, consent to the survey	**Outing day and behavior of that day (location/vehicle),** timeframe, **length of stay**, indoors/outdoors, whether mask was worn or not, number of people contacted, types of persons contacted, **objects with which respondent came into contact,** things done for infection prevention

Table S2. Occupation categories and number of subjects

Occupation/industry	n
Agricultural/forestry	1
Fishery	0
Mining, quarrying, gravel-digging	0
Construction	49
Manufacturing	159
Electricity/gas/heat/water	6
Information communication	81
Transportation/postal services	52
Wholesale/retail	106
Finance/insurance	68
Real estate/leasing	46
Academic research (e.g., research laboratory)	1
Specialized services (e.g., law offices)	19
Technical services (e.g., design, advertisement, consulting)	19
Accommodation and/or food/drink services (e.g., hotels, restaurants)	27
Living-related services (e.g., hairdresser, laundry)	13
Other living-related services (e.g., travel, ceremonial occasions)	8
Entertainment (e.g., leisure, sports)	10
Education/learning-support (e.g., schools, libraries, cram schools)	69
Medical/welfare services (e.g., hospitals, nursing care)	62

Compound services (e.g., postal, co-op)	7
General services (things not categorized in other categories, such as employment referrals, security, waste disposal)	113
Public services (e.g., public office employment, excluding those that belong in other categories)	28
Industry other than those listed above (i.e., uncategorizable)	49
Homemaker	171
Student	20
Unemployed/not working currently	88
Total	1,272

1.2 Information retrieved from surveys

Table S3 shows the aggregated target data by age group, residential area, and occupation. Sex was divided into male and female and age into five-year groups (covering 20 to 69 years of age). Yes/no categorizes whether or not the subject is employed, with 'non-employed persons' referring to those who responded as homemakers, students, or unemployed. Although this survey obtained information regarding Tokyo/Kanagawa prefecture residence, existence of occupation, and sex, such information was not used in the analysis. Of the 1,288 aggregation targets, 10 subjects who responded with responses other than the locations in question, two who used vehicles such as airplanes or motorbikes, and anyone who did not write contact objects in their responses were excluded. This process of elimination resulted in a final analysis of 1,260 subjects. Table S4 shows the locations where the participants spent the most time, as well as the number of people with whom they spent time during the research period.

Table S3. Retrieval numbers by age group, residential area, and existence of occupation

			20s–30s				40s–50s				60s and over				Total
			Tokyo		Kanagawa		Tokyo		Kanagawa		Tokyo		Kanagawa		
			Yes	No	Yes	No	Yes	No	Yes	No	Yes	No	Yes	No	
Preliminary survey	11/27–11/30, 2020	Numbers distributed	113,977				22,226				7,261				143,464
		Numbers retrieved	3,020				2,337				1,420				6,777
		Consenting subjects	242	55	160	43	438	85	350	64	214	95	121	76	1,843
Main survey	12/3–12/9, 2020	Numbers distributed	239	53	158	43	415	73	332	49	197	91	113	65	1,828
		Numbers retrieved	200	42	128	35	336	64	290	40	195	71	100	55	1,536
		Aggregated targets	157	35	109	30	276	59	247	39	134	65	85	52	1,288

Table S4. Outing behavior (location/vehicle) and number of people between December 3 (Thursday) and December 7 (Monday)

Type	Item	Number of people
Location	School/university	18
	Workplace	127
	Client workplace	75
	Hospital	119
	Gym	83
	Restaurant	140
	Bar	59
	Theater/cinema	45
	Supermarket/CVS	300
	Department store	102
	Amusement park	10
	Hotel	42
Vehicle	Train	84
	Bus	26
	Taxi	30
Total		1,260

CVS, convenience store

2. Supplementary analysis

2.1 Analysis of significant differences using the Tukey-Kramer test

Figure S1 presents multiple comparison heat maps using the Tukey-Kramer test which was conducted as a reference. The Tukey-Kramer test is a one-step multiple comparison procedure, while the Scheffé test is applied to a series of estimates of all possible contrasts between means at the element level, and not just the difference in paired comparisons. For this reason, the Tukey-Kramer is generally more likely to produce significant differences. Although the Scheffé test was used in this study, the results of the Tukey-Kramer test are also provided as additional reference information in the Supplementary Information section.

Since significant differences were found between hotels and all other places except for amusement parks and schools/universities, similar to the findings of the Scheffé test, it could be concluded that hotels have a higher number of contact objects compared to other locations. In addition to the Scheffé test, the results demonstrated that supermarket/CVS had a significantly lower number of contact objects compared to client workplaces and restaurants. Conversely, no significant difference between vehicles was found in either the Scheffé or Tukey-Kramer test.

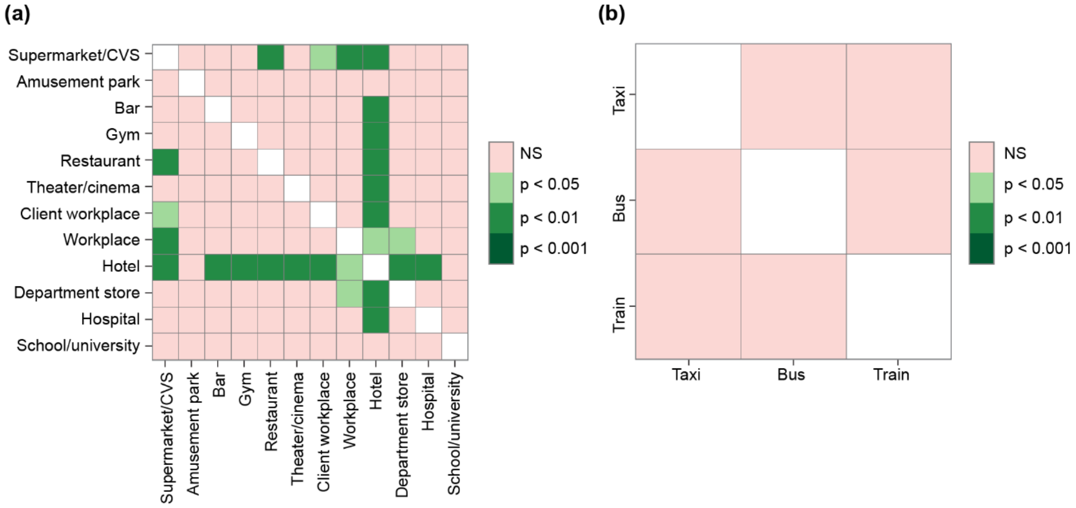

Figure S1: Heat maps of multiple comparisons of the number of contact objects for (a) each location and (b) each vehicle based on the Tukey-Kramer test.

2.2 Contact objects by components

An object may consist of more than one component. For example, a door is composed of the actual door and its doorknob, and a car has many areas that can be touched (e.g., handles, doors, and gear levers). Thus, the same object may have different 'frequently touched' points depending on the number of individual components. Figure S2 shows the top 15 items by component. The small graph set into Figure S2 is a histogram (bins: 50) of the frequency distribution of the objects.

Doors, the most frequently touched objects, consist of levers, handrails, and/or doorknobs/buttons. Of these components, doorknobs were touched 357 times, and the buttons of automatic doors were touched 39 times. Elevator buttons were touched 202 times, followed by sink faucets and escalator handrails. Similar to Figure 2, the histogram has a structure in which objects with a lower appearance frequency occupy the majority of all types. It should be noted that although chairs and baskets, which had a high contact frequency (Figure 2), are composed of several components, they do not appear at the top of the component list, as they often appear as chairs and/or baskets.

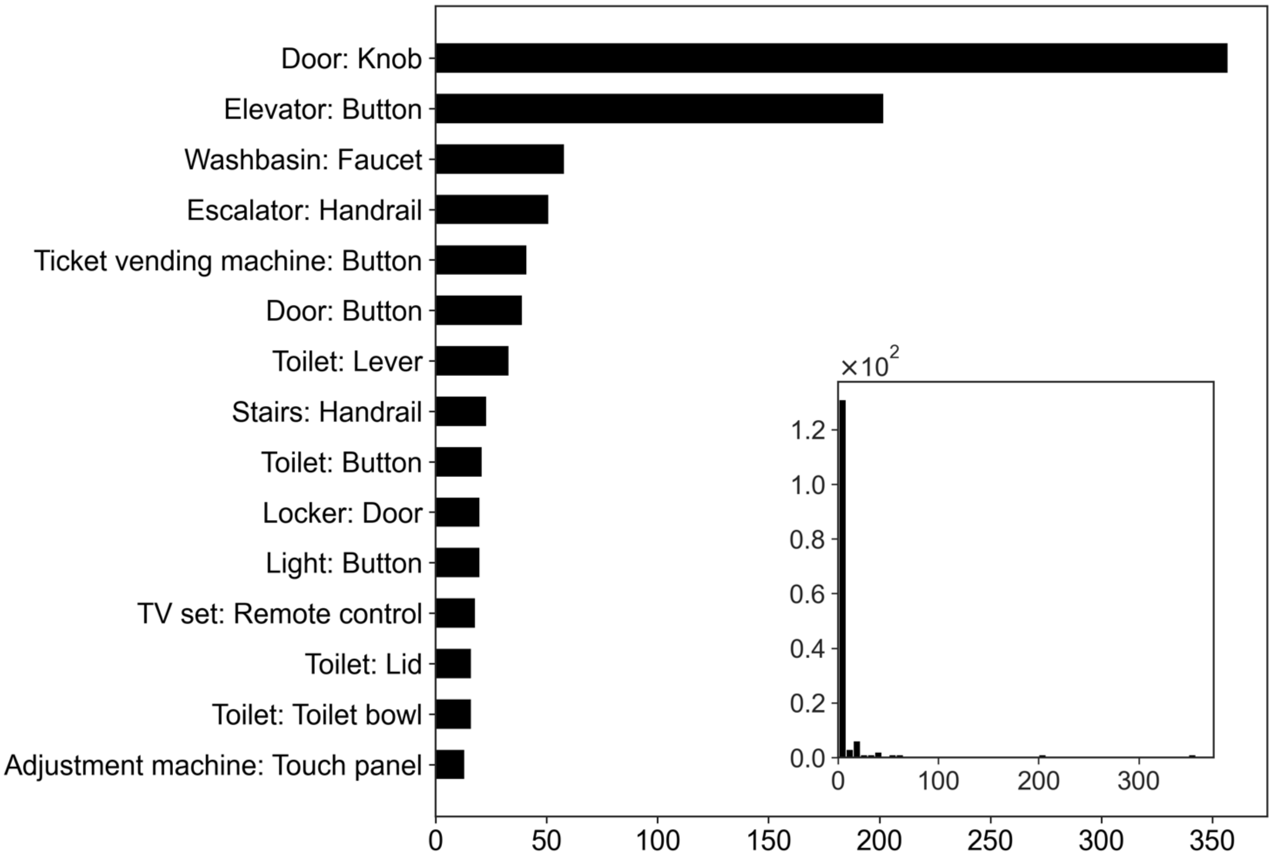

Figure S2: Distribution of the 15 most touched object components. There were 148 types of components, and a total of 1,204 components were touched. In the histogram (bins: 50), 65 types of components have an appearance frequency of 1, and 45 types of components have an appearance frequency of 2. These findings present a structure in which components with a small appearance frequency account for the majority of all appearance frequencies.

2.3 Number of users and objects per location/vehicle

Figure S3 shows the number of users and objects with which the participants came in contact at a given location. Supermarket/convenience store (CVS) was the location with the highest number of users, e.g., 300 users, 218 types of objects, and 1,336 objects in total were registered. In comparison, restaurants had 140 participants, and workplaces had 127 participants. Not only were taxi, bus, and amusement park reported by fewer users, the total number of types and contact objects were also found to be smaller than those in the aforementioned list. The overall trend was that the numbers and types of contact objects increased linearly as the number of users increased. As this graph suggests, more users lead to more contact objects and object types.

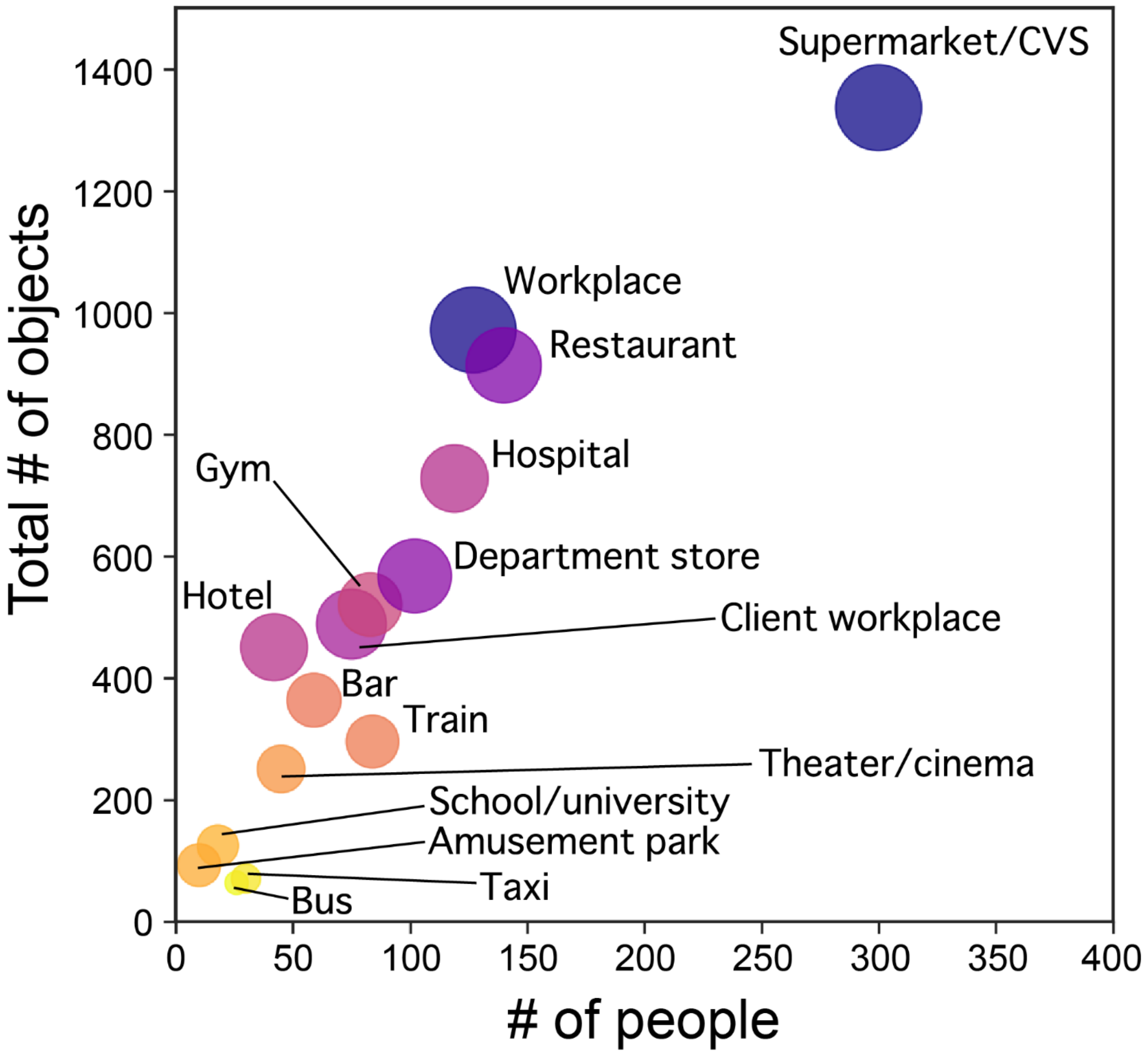

Figure S3: The number of users per location and the number of objects touched in each location. The size of the circles in this figure represents the number of object types with which subjects came into contact in the corresponding location.
CVS, convenience store

2.4 Length of stay

The data acquired in this study consisted of an item in which respondents wrote down the length of stay at each location or vehicle. The length of stay was divided into 11 timeframes: "under 15 min," "15–30 min," "30–45 min," "45–60 min," "1–1.5 h," "1.5–2 h," "2–2.5 h," "2.5–3 h," "3–4 h," "4–5 h," and "6 h or more." Of the 1,260 respondents, only 886 answered this question regarding their length of stay. Figure S4 shows the number of contact objects according to the 11 length-of-stay categories. The size of the dot represents the number of people who came into contact with the object during a given period (i.e., length of stay). For example, the figure shows that 28 subjects touched one object for a duration of less than 15 min. The correlation coefficient between the length of stay and the object touched was small at 0.23, and no correlation between the length of stay and the number of contact objects was found.

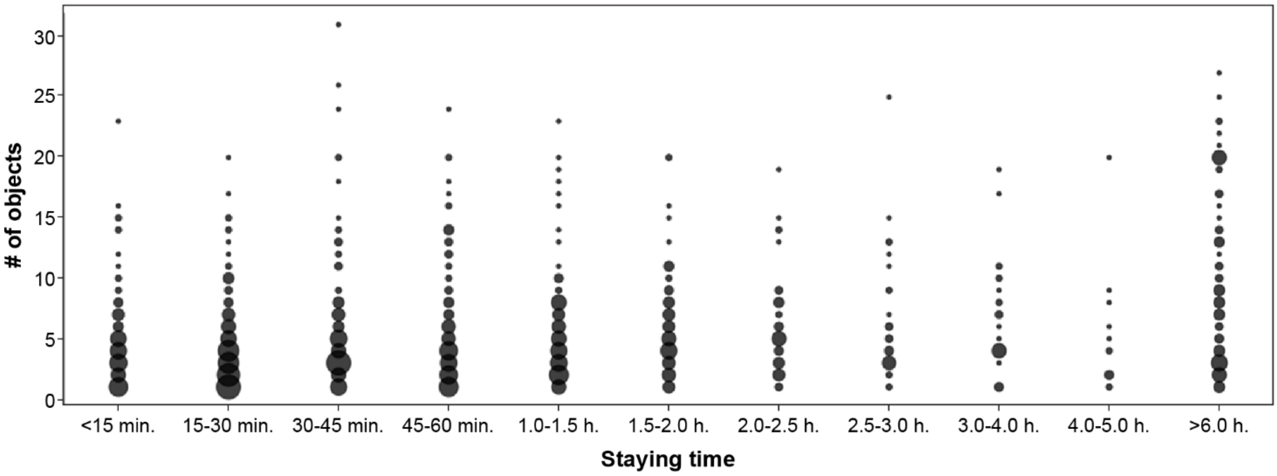

Figure S4: Number of contact objects according to the 11 length-of-stay categories. The size of the dot represents the number of people who came into contact with an object during a given period, i.e., the length of stay. The greatest number of contact objects was 31 for a duration of 30–45 min. Moreover, fewer objects were touched in locations where the participants stayed for more than 6 h.

2.5 Restroom and door contact

Regarding the appearance frequency of restrooms in relation to the number of users at each location or vehicle, the appearance frequency of restrooms as a contact object at supermarkets/CVS, train stations, and client workplaces was found to be very small (Figure S5). Hotels, however, had the highest contact ratio with restrooms, whereas schools/universities, bars, theaters/cinemas, and workplaces also had high ratios. The contact ratio of hospital restrooms, which were reported in several previous studies, was small compared to other locations. However, hospitals generally have a higher rate of infection than other locations; therefore, PCR testing and disinfection are necessary, even if the contact ratio is small. Notably, hotels, schools/universities, bars, theaters/cinemas, and workplace restrooms have rarely been mentioned in previous studies. Therefore, PCR tests and disinfection at these locations should be examined more extensively in the future.

The reason for the higher contact ratio for restrooms in bars compared to restaurants is likely due to the high frequency of use as a result of alcohol consumption. Similarly, the contact ratio in workplaces was higher than that in client workplaces, which is believed to be due to an individual's general length of stay.

In all locations, except for supermarkets/CVS, doors were found to be the object with the highest contact frequency. Doors are also the object with which everyone who enters or leaves a place comes into contact. The contact ratio of hotel doors was the highest, followed by workplaces, client workplaces, and hospitals (Figure S6). Hotels, where the number of contacts with doors was the highest in relation to the number of users, had many doors (e.g., to guest rooms, entrances, restrooms, and bathing facilities). Various door types were also reported in workplaces and client workplaces (e.g., entrances, warehouses, restrooms, reception rooms, lockers, and emergency exits). In hospitals, doors for spaces such as entrances, examination rooms, and restrooms had high appearance frequencies. Since a door is an entity that is used to cross between spaces, it is guaranteed that it will be touched by many people. Therefore, the doors at these sites should be prioritized for disinfection.

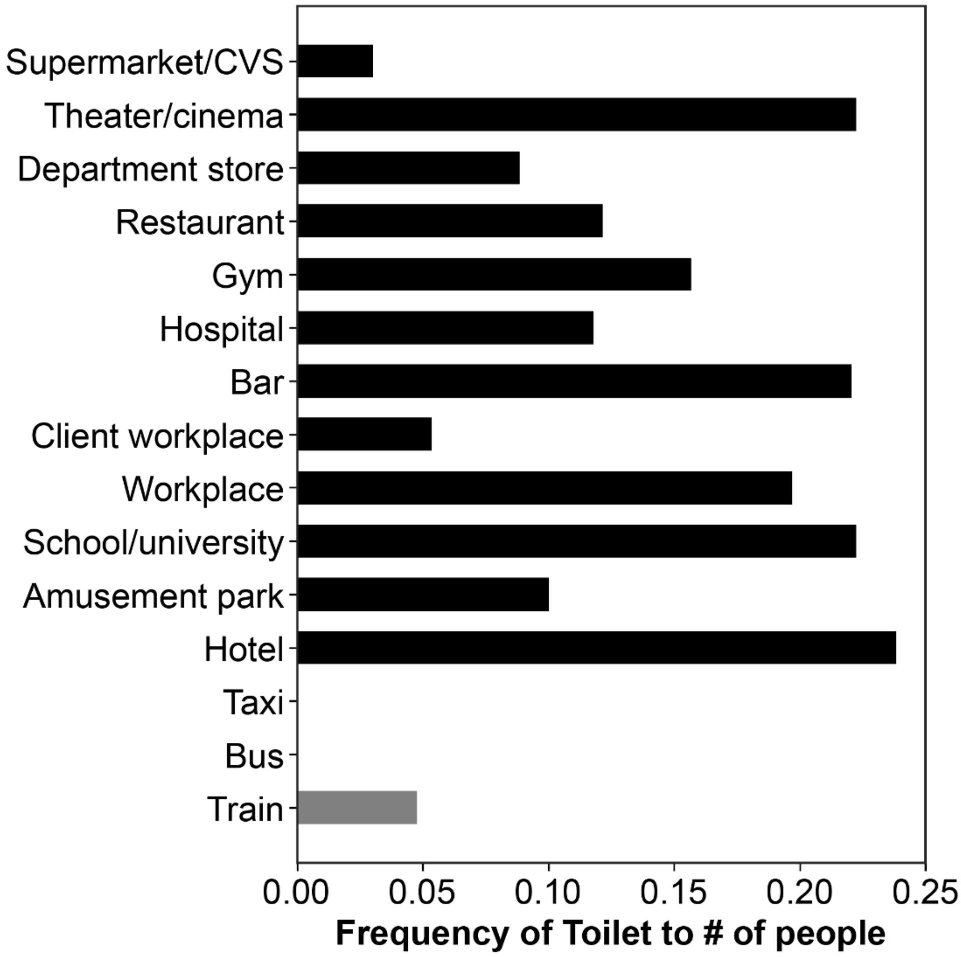

Figure S5: Number of toilet appearances to the number of users for each location/vehicle. The number of appearances of restrooms, as a contact object, was extremely small for supermarkets/CVS, trains, and client workplaces. Conversely, the largest number of restroom appearances was reported for hotels followed by schools, universities, bars, theaters/cinemas, and workplaces.
CVS, convenience store

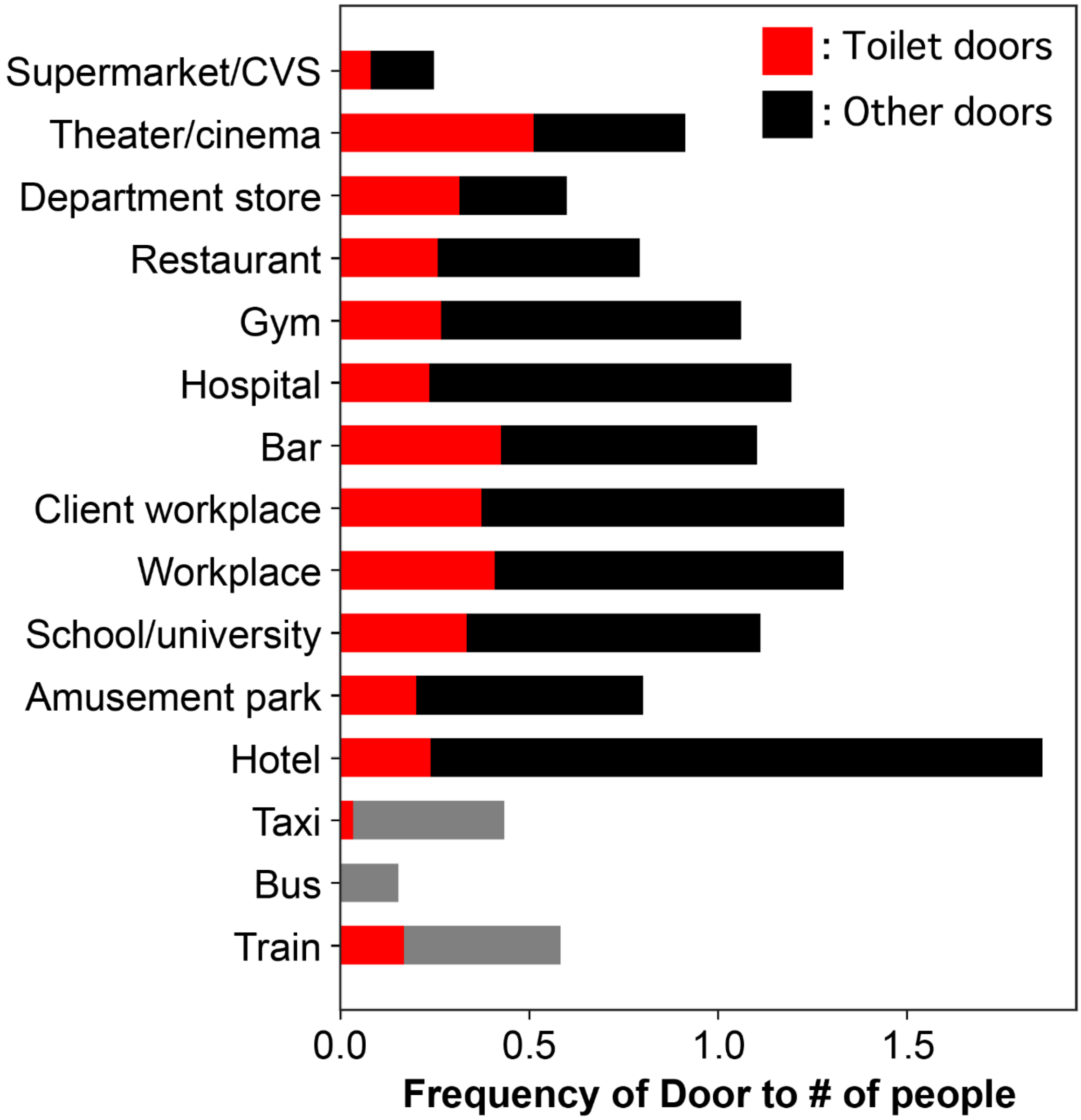

Figure S6: Appearance frequency of doors and toilet doors to the number of users for each location/vehicle. To compare these results with the discussion of the results presented in Figure S7, restroom doors are also presented for reference. An accurate comparison in this regard is difficult, however, since there are overlaps with "space" and "location," as mentioned in the Study Design section. As an entity, restroom door was also clearly described and recorded as {space: toilet, object: door}. Therefore, the toilet "space" was described as "toilet" and/or "other." However, some restrooms do not have entrance doors (e.g., public restrooms and restrooms in a building). In such cases, it was deemed suitable for the restroom doors to be viewed as functioning solely for reference purposes.
CVS, convenience store
